# “Unfortunately, we can't avoid everything”: a qualitative study of municipal mental health workers' experiences with workplace violence

**DOI:** 10.1080/17482631.2026.2741483

**Published:** 2026-11-03

**Authors:** Hilde Karlsen Sanna, Marit Helene Hem, Ottar Ness, Tine Kristin Grimholt

**Affiliations:** a Centre for Diaconia and Professional Practice, VID Specialized University, Oslo, Norway; b VID Specialized University, Oslo, Norway; c Norwegian University of Science and Technology, Trondheim, Norway

**Keywords:** Community-based care, focus group interviews, occupational health, reflexive thematic analysis, violence prevention, workplace violence

## Abstract

**Purpose:**

The experiences of municipal mental health workers involving workplace violence remain underexplored. This study aimed to obtain an in-depth understanding of how mental health workers perceive and manage challenging encounters with service users.

**Methods:**

We conducted six focus group interviews with 38 participants from urban and rural municipalities and private, nonprofit organisations in Norway. The interviews were analysed using reflexive thematic analysis.

**Results:**

Workplace violence is a complex and multifaceted challenge within municipal mental health services. We identified four main themes (1) Unpredictable situations perceived as precarious and distressing, (2) Unclear and uncertain collaboration and responsibility distribution, (3) Risk reduction through a dynamic approach based on relationships and knowledge, and (4) Confidence to cope with challenges developed within the workplace.

**Conclusions:**

Workplace violence needs to be addressed in municipal mental health services. Mental health workers perceive unpredictability over time as equally stressful as being exposed to violence. To ensure safe workplaces providing high-quality mental health services, systematic collaboration and clearer distribution of responsibilities across various agencies and healthcare levels are required. Knowledge development about consequences and strategies for preventing and managing workplace violence in the context of municipal mental health services is vital.

## Introduction

Workplace violence in healthcare settings has been increasing (Choi et al., 2023; Mento et al., 2020). The likelihood of being subjected to violence is higher in mental health services than in other healthcare systems (Dean et al., 2021; Edward et al., 2016): 40–65% of mental health workers have been exposed to such violence (Fujimoto et al., 2017; Konttila et al., 2020). Workplace violence involves aggressive actions and behaviours, perceived as humiliating and frightening by those who are exposed (Mento et al., 2020; Zhong & Shorey, 2022). Incidents include verbal aggression, sexual harassment, and physical attacks (Yang et al., 2018). Workplace violence can have severe consequences (Konttila et al., 2020; Mento et al., 2020; Tonso et al., 2016) and lead to physical and psychological burdens, including sick leave, burnout, and compassion fatigue (Fujimoto et al., 2017; Mento et al., 2020; Tonso et al., 2016). This has implications for the workforce and society (Liu et al., 2019; Mento et al., 2020) and can contribute to poorer treatment outcomes for service users and reduce the quality of care (Galinsky et al., 2010; Roche et al., 2010; Zhong & Shorey, 2022).

Internationally, mental health services have been undergoing deinstitutionalization (Campbell, 2017; Konttila et al., 2020; Maagerø-Bangstad et al., 2019; Ose & Kaspersen, 2023). Service users previously treated in hospitals increasingly receive municipal mental health services. This may affect the incidence of workplace violence and the working conditions of mental health workers outside hospitals (Parr & Cetrano, 2024). Although recent research has begun to address workplace violence in community mental health services, knowledge remains limited regarding how municipal mental health workers experience and manage challenging encounters with service users (Choi et al., 2023; Parr & Cetrano, 2024).

Professional and political principles emphasise the importance of a person-centred approach in mental health services (Hummelvoll et al., 2015; McCormack & McCance, 2017; Ministry of Health & Care Services, 2023; Ose & Kaspersen, 2023). Despite improvements in treatment interventions, including reduced use of coercion, enhanced rehabilitation services, and pharmacological treatment developments, workplace violence has not decreased (Flannery & Flannery, 2023; Flannery et al., 2018). Addressing this requires sufficient human and financial resources (Rimondini et al., 2019). Furthermore, the health and well-being of mental health workers have not been the focus, despite the potential negative consequences for employees, employers, and service users (Morse et al., 2012).

Few mental health service users are at risk of behaving violently, and mental illness is not in itself associated with an increased risk of violence (Ose et al., 2017). Violence perpetrated by service users is often associated with their personal characteristics, and the contextual factors and circumstances that may contribute to inappropriate ways of being are seldom considered (Haugvaldstad & Husum, 2016). The causes of violence are rarely described (Løvsletten et al., 2020; Sowislo et al., 2017). Torrey (2011) links violence from service users to low socioeconomic status and inadequate treatment of mental health issues in municipal mental health services. Additionally, positive symptoms of schizophrenia, poor compliance, and substance use are associated with an increased risk of violence (Ose et al., 2017). Nonetheless, workplace violence research has focused on hospitals and inpatient settings, indicating insufficient knowledge concerning municipal mental health services (Campbell, 2017; Fujimoto et al., 2017; Konttila et al., 2020; Maagerø-Bangstad et al., 2020).

### Context of the study

This study explored how municipal mental health workers in Norway experienced workplace violence. Mental health workers are a heterogeneous group with various professional backgrounds. They are assistants without formal education, skilled workers and college graduates, with and without further education. In this study, we use the term mental health workers to employees with formal health and/or social work education.

**Table I.** Description of the focus groups.

Conduction of focus groups, included description of municipalities/organisations and participants						
Focus group	1	2	3	4	5	6
Duration of interview	1 h, 48 min.	1 h, 42 min.	1 h, 40 min.	1 h, 21 min.	1 h, 28 min.	1 h, 45 min.
Type of municipality and organisation	Rural municipality	Nonprofit organisation	Rural municipality	Urban municipality (small city)	Nonprofit organisation	Urban municipality (a part of a big city)
Number of participants	8	8	6	5	5	6
Gender	8 women	7 women 1 man	5 women 1 man	4 women 1 man	2 women 3 men	5 women 1 man
Approx. age	25–60+	25–60+	40–60+	25–50+	25–55	25–60
Education (FE = further education)	1 skilled worker 2 skilled workers FE 1 social educator 1 nurse 2 nurses FE 1 social worker FE	1 skilled worker 2 skilled workers FE 3 nurses 1 nurse FE 1 social educator FE	2 skilled workers FE 1 social educator 2 social educators FE 1 clinical social worker	1 skilled worker 2 skilled workers FE 1 public health 1 nurses FE	2 skilled workers 2 nurses 1 public health	2 skilled workers 1 social educator 2 nurses 1 criminologist
Experience of practice	2–30 years	5–25 years	13–30 years	1–20 years	1–8 years	1–22 years

**Table II.** Example from the analysis process.

Codes	Initial themes	Themes
Always on standby Looking over your shoulder	Insecurity is worse than concrete violence	Unpredictable situations perceived as precarious and distressing
Unsafe in your own home Uncertainty about what may happen	Impact on an emotional and private level	
Unattainable expectations Disagreement on mandate	Different understandings and varied cooperation between health levels and agencies	Unclear and uncertain collaboration and responsibility distribution
You have to argue for help Depends on who you meet	It depends on the person what help you get	
Knowledge is not enough You have to read the situation	A dynamic approach based on knowledge and understanding	Risk reduction through a dynamic approach based on relationships and knowledge
We know them well Relationships evolve over time	Relationships over time provide motivation and “direction”	
Knowing that someone has your back Collaboration with colleagues means everything	A sense of community and supportive leadership as safety factors	Confidence to cope with challenges developed within the workplace
Prevention is continuously focused Training systematised/not systematised	Training systems for prevention and management differ in range and systematics	

Over years, municipalities have been assigned additional tasks and responsibilities because of organisational changes in the mental health system. In addition to decisions pursuant to the Health and Care Services Act. Act No. 30 (2011), service users may be subject to “compulsory outpatient treatment” (Mental Health Care Act. Act No. 62, 1999), and/or court orders to transfer to compulsory mental health care (The Penal Code & Act No. 28, 2005). Individuals are considered both “patients” in the specialised mental health services and “service users” in the municipal mental health services (Kisely et al., 2017; Løvsletten et al., 2020), which can lead to complicated interactions. In this study the term “service user” refers to the individuals’ mental health workers encounter in their work, as recipients of municipal mental health services.

The municipalities are responsible for providing proper mental health services to all residents in the municipality (Health & Care Services Act. Act No. 30, 2011), and there is an increased need for qualified staff (NOU 2023:4, 2023; Ose & Kaspersen, 2023; Øygard et al., 2020). In Norway, municipal mental health services are not uniformly organised, and the municipalities include populations with varied demographics and economic statuses. As employers, municipalities are required to ensure a ‘fully adequate’ working environment that safeguards the physical and mental health and welfare of employees, according to the Working Environment Act. Act No. 62 (2005). Providing sound staff training and education is necessary. To fulfil the stated goals of retaining and recruiting staff and expertise (Ministry of Health & Care Services, 2023), and to build services that prevent and manage workplace violence, competence development is required (Hagen & Svalund, 2019; Maagerø-Bangstad et al., 2019; Maagerø-Bangstad, 2024).

Although the number of municipal mental health service users exhibiting violence directed at mental health workers is low (Ose et al., 2017), the consequences can be serious; thus, mental health workers’ experiences of workplace violence should be explored (Fujimoto et al., 2017; Liu et al., 2019; Mento et al., 2020; Tonso et al., 2016). This study specifically addressed workplace violence in municipal mental health services. By exploring the experiences of mental health workers, we sought to develop an in-depth and nuanced understanding of how challenging encounters with service users were perceived and managed. The research questions were as follows: (1) How do mental health workers experience workplace violence? (2) What strategies do mental health workers employ in challenging situations?

## Materials and methods

We chose a qualitative design to gain access to mental health workers’ experiences and understand workplace violence in municipal mental health services. Given the limited research available, a descriptive and exploratory approach was appropriate (Kvale & Brinkmann, 2009). This study was grounded in hermeneutic phenomenology, which aims to describe participants’ lived experiences (Cresswell & Poth, 2018; Drageset & Ellingsen, 2010). We strove for reflexivity about our preconceptions and sought to understand the individual elements in relation to the whole, and vice versa, by engaging with participants’ narratives and contexts. By remaining open and allowing ourselves to be affected by participants’ experiences, we aimed to better understand their perceptions and lived experiences.

### Study participants and recruitment

We employed strategic sampling to ensure a broad variety of employees in Norwegian municipal mental health services. We included urban and rural municipalities and private, nonprofit organisations providing 24-hour staffed housing. Municipalities often purchase services for service users because of complex challenges (Hansen et al., 2023).

The first author contacted managers from ten different units; six agreed to participate. Managers distributed information letters to employees who were willing and able to participate in the scheduled focus groups. To ensure a diverse sample, we sought both men and women, as well as variations in age, education, and experience (Halkier, 2012) (Table I).

### Data collection

Focus group interviews were chosen instead of individual interviews because interaction among participants could elicit shared, contrasting, and developing accounts of workplace violence, providing insight into how experiences and practices were collectively understood and negotiated (Krueger & Casey, 2014; Malterud, 2012). Nuanced and varied descriptions were facilitated through interactions and discussions within the groups. Focus groups also allowed us to revisit topics when new contributions indicated a need for further elaboration. We sought breadth and nuance rather than consensus, focusing on participants’ reasoning about their experiences (Halkier, 2012).

We conducted six focus group interviews with 38 participants (seven men and 31 women) between June and December 2023. The interviews ranged from 1 h 21 min to 1 h 48 min. The inclusion criteria were health and/or social work education, a minimum of one year of practice, and at least a 50% permanent position at the respective workplace. The focus group interviews were conducted at various workplaces in calm and familiar environments. This setting facilitated the exploration of social norms and participants’ experiences with workplace violence (Krueger & Casey, 2014). Four individuals conducted the six interviews. The first author moderated five interviews. The sixth interview was conducted at the first author’s workplace, and an external researcher moderated it to ensure analytical distance and reduce the potential influence of the first author’s familiarity with participants and the setting. The first author co-moderated this interview. The external researcher also co-moderated another interview, and the last author co-moderated one interview. The remaining three interviews were co-moderated by an external person familiar with conducting focus group interviews. The interviews were recorded, and the first author transcribed them verbatim.

A semi-structured interview guide containing open-ended questions was used (Cresswell & Poth, 2018; Kvale & Brinkmann, 2009). It encompassed six themes: experiences with workplace violence, frameworks, competence, service provision, personal thoughts and perceptions, and collaboration. All interviews started with an open-ended question regarding whether participants had experienced workplace violence and whether they would be willing to share their thoughts. Follow-up questions (“Can you tell us more about ...?” or “Can you give specific examples?”) were posed to explore the topics and ensure understanding (Halkier, 2012).

An advisory board consisting of five people (two mental health workers, one peer consultant, and two next of kin) participated in all phases of the study, from developing the interview guide to discussing the analysis and interpretation of the findings. The advisory board contributed experiential and professional perspectives that broadened the research team’s reflections.

### Analysis

The interviews were analysed using reflexive thematic analysis (Braun & Clarke, 2021, 2022, 2025). The first author is a nurse with extensive experience in municipal mental health work; thus, we aimed for reflexivity throughout the process. Before and during the analysis, the first author documented reflections in a research diary to maintain a critical distance from her own and participants’ perspectives. Moreover, the research team comprised two psychiatric nurses and professors in mental health (the second and last authors), and a family therapist and professor of counselling (the third author). This diversity of expertise of the research team and the advisory board helped explore multiple viewpoints and enhanced data interpretation.

After each interview, the first author and the moderator/co-moderator discussed the interview situation and group dynamics and identified areas requiring adjustment. The first two interviews were transcribed before the third interview, enabling the interview guide to be refined for the subsequent interviews.

The analysis was conducted in six phases. In phase one, the first author became familiar with the data through repeated listening to the recordings and reading the transcripts, while the author team discussed initial impressions and preliminary patterns. In phase two, the first author manually coded the transcripts in Microsoft Word and organised the codes in Microsoft Excel; no dedicated qualitative data analysis software was used. She highlighted words, sentences, and longer passages relevant to the research questions and assigned initial semantic codes. Some extracts were assigned more than one code when they conveyed different meanings. In phase three, the codes were compared, revised, and grouped into 18 provisional clusters based on shared patterns of meaning. These clusters informed the development of candidate themes. In phases four and five, the candidate themes were reviewed, developed, and named. Throughout this process, the first author moved iteratively between the coded extracts and the complete dataset. The developing codes, clusters, and themes were discussed with the co-authors, who contributed by questioning assumptions and suggesting alternative interpretations. In phase six, the codes and theme development were considered again in relation to the complete dataset and the research questions. The analysis resulted in four interrelated themes (Braun & Clarke, 2022) (Table II).

### Research ethics

This study was approved by the Norwegian Agency for Shared Services in Education and Research (reference number 387616) and assessed by the Regional Committees for Medical and Health Research Ethics (REC) in Norway (reference number 569805). In accordance with the Norwegian Health Research Act. Act. No. 44 (2008), studies that do not involve patients do not require approval by REC. Nevertheless, an enquiry was submitted to REC prior to initiating the project and as the study was considered to fall outside the scope of Health Research Act. Act. No. 44 (2008), a full application was not required. According to the Declaration of Helsinki all participants received written and verbal information about the study’s purpose and their rights, including anonymity, confidentiality, and the option to withdraw without any consequences (World Medical Association, 2013). Written consent was obtained. To ensure voluntariness, especially since one of the interviews was conducted at the first author’s workplace (Coghlan & Casey, 2001), the participation invitation was distributed through the department manager, and participants were informed that the interview would be led by an external moderator. The names of the individuals and workplaces mentioned in the interviews were removed or altered in the transcriptions to ensure anonymity. Participants were informed that the first author would send a follow-up text message after a few days and could be contacted if they required a conversation following the interview.

## Results

The analysis identified four themes that helped explain participants’ experiences of workplace violence and what strategies they employ in challenging situations: (1) Unpredictable situations perceived as precarious and distressing, (2) Unclear and uncertain collaboration and responsibility distribution, (3) Risk reduction through a dynamic approach based on relationships and knowledge, and (4) Confidence to cope with challenges developed within the workplace. Participants were assigned pseudonyms. M and CM denote the moderator and co-moderator, respectively.

### Unpredictable situations perceived as precarious and distressing

The first theme illuminated how participants were affected by their experiences with workplace violence. They discussed various forms of violence, noting that it was not necessarily physical assaults or actions that caused physical pain that were perceived as the most distressing. The unpredictability and being “on standby” were demanding, as they lacked control over what could happen in their work.

Lene (FG6): Violence also applies to psychological violence.

Berit: That uncertainty and ambiguity ... It’s almost worse than an outburst, in my impression, because you’re constantly looking over your shoulder, right?

Several examples of how safety was threatened owing to unpredictability were discussed. Participants described experiences of not knowing what they would encounter when visiting service users in either co-located or more independent housing. Additionally, this unpredictability affected their private lives, particularly in smaller municipalities, where participants often lived and worked in the same community. However, participants did not elaborate on how they were affected beyond making statements like “you can feel it” or “it did something to me.”

Ine (FG1): And then there is the feeling of being unsafe in your own home. I got a text message from a colleague because he (the service user) had said that he was going to use the knife on me (...) and then I felt that I lost my sense of safety [long pause and complete silence around the table]. I went up several times to check doors and things, and it did something to me in the long run.

Ine elaborated that living in the local community created an additional burden because it was a small municipality, and her residence was known to most people. A similar situation was described by another participant who experienced a form of “invasion” of her private life when a service user’s statements and behaviour affected her family members. The participant expressed concerns regarding her inability to influence the situation, particularly when the service user indicated that her family did not exist, alongside statements of future violent acts. The challenges posed by such information to the participants and colleagues who were aware of these threats were discussed. Susann (FG1), who experienced the threat, mentioned feeling powerless: “I’ve been sitting at home and the police are calling, ‘Are you safe?’ they ask ... Then I sit alone with my children and ... I don't know if I’m safe ...” This quote highlights a lack of control in a highly unpredictable situation and that not having opportunities to solve the problem was distressing. Similar to Berit (FG6), Susann (FG1) expressed the following: “I think it would have been easier if it had been like, ‘Now I’m going to get you.’ (…) It’s kind of lurking there all the time, and I don’t know how far he’ll go.”

Participants described some risk of violence as unavoidable and as an inherent part of their work. The exchange between Erik and Tor illustrates this: Erik (FG5): ‘It resulted in a “beating” in a way.’ Tor: ‘Yes, and unfortunately, we can’t avoid everything.’ Other participants referred to such risk as ‘the name of the game’ or as something they had become accustomed to.

### Unclear and uncertain collaboration and responsibility distribution

Participants discussed various aspects of the collaboration and division of responsibilities between other healthcare levels and agencies and that they sometimes felt alone in their efforts to provide support to service users with complex challenges. Amid serious situations, other agencies providing assistance and other parts of the healthcare system accepting responsibility appeared crucial. However, participants believed that this was complicated owing to the differing perspectives among various healthcare levels regarding service users’ needs. One participant described a threatening situation arising from a service user’s admission/hospitalisation request.

Ine (FG1): He just showed that he had a knife, simply to be heard, in a way ... He had several admissions due to paranoid fear, but he wanted more help than a quick trip to the hospital (...). This was when he started with the knives.

This situation illustrates how pressure on available inpatient beds, together with differing views across healthcare levels and agencies about how service users’ needs should be met, may have contributed to an escalation of the situation. Participants also described having to advocate for service users to be assessed by, for example, emergency services. There was considerable variation in how participants experienced collaboration with other agencies. Collaboration was perceived as depending as much on individuals as on the system. Uncertainty about whether assistance would be available in precarious situations was interpreted as an additional burden.

Mari (FG4): What I find most frightening is (...) when we try to admit someone voluntarily and we encounter resistance, then we can’t do our job. What’s the point if we can’t proceed because we face opposition?

Participants expressed that collaboration could be challenging because they did not have decision-making authority. Although they communicated their observations to emergency services, if no measures were taken, this could lead to an increased risk, as service users might feel rejected, and the situation could escalate. Participants also highlighted positive collaboration with other agencies; for instance, security services or the police could assist in acute situations that participants could not manage. Large geographical distances were also noted as a challenge, as assistance may take a long time to arrive. Else (FG6) said:

You can call the security service in the building, and that provides extra security, and then there are the emergency services, like the police and ... But, if someone is standing at the door ringing the bell, you really don’t want to open up and talk to them if you’re alone and it’s two o’clock in the morning … It becomes a judgement call for the person who is there, alone.

Working alone was described as something to be avoided. Nevertheless, participants felt that this was unavoidable in municipal mental health services offering 24-hour mental healthcare.

### Risk reduction through a dynamic approach based on relationships and knowledge

Relationships with service users and knowledge were identified as central to managing challenging situations. Getting to know service users over time and understanding their behavioural patterns and reactions provided a sense of safety, although participants did not perceive this knowledge as entirely predictive. Hege (FG1) highlighted this experience of underlying uncertainty: “We know them so well here, some for many years, but still ... We may think we might know, but we cannot predict what is going to happen.”

Participants also discussed the importance of relationships in enabling them to contribute to changes in service users’ situations. They focused more on service users’ possibilities than on their limitations and sought to create opportunities for service users to develop skills and manage new situations. This depended partly on individual mental health workers’ experience and their confidence in managing unpredictable situations.

Erik (FG5): As the relationship improved and we felt that the outburst isn’t so hard that we can’t stand it, then (...). From experience, we see that if we don’t react to his rapid movements, things calm down quickly. So, we have begun to “take a chance,” so to speak, and not react so much to his movements.

This statement illustrates participants’ ability to adjust their approach as they became acquainted with the service user. They made choices to face potentially risky situations to facilitate the service user’s mastery in challenging situations. Time was emphasised as important for relationship building. The conversation continued:

M: You take a calculated risk, essentially?

Erik: Yes, and we adjusted it a bit according to the relationship. If you feel safe, you can sit almost alone, with one staff member three or four metres away, while at other times, we might need to sit with two people next to each other.

Tor: But it must be people who know him well [mmm ... around the table]. If not, it will probably just get worse.

Erik: Yes, because if you “jump” at his reaction, you’ll get a counterreaction, and then it escalates.

Participants also discussed how knowledge and professional understanding were crucial for acting in a dynamic way. They reported often “reading the room” and using their “clinical gaze” before almost instinctively making decisions about their approach. This provided them with a “hunch” when something was brewing and allowed them to maintain their safety through their response. Participants also discussed how they “lowered their guard” during more stable phases, allowing them to “forget” the more demanding periods. Their familiarity with service users’ histories of violence along with an understanding of the possible causes of violent actions appeared to contribute to a dynamic way of working. This also included working in pairs during particularly demanding situations.

Nina (FG4): There are also places where (…) you don’t know what might happen when you get there.

Mari: If there has been a lot of weapons and stuff in the area, then we have gone in pairs.

Per: It becomes an ongoing assessment we make then, related to the service users.

Participants highlighted the complexity they experienced in municipal mental health services. Factors other than service users could pose risks to staff and the surrounding environment. The option of visiting in pairs appeared crucial to participants’ ability to manage such situations.

### Confidence to cope with challenges developed within the workplace

Participants described explicit expectations for themselves and for collaborative team efforts. They also discussed service changes characterised by increasing pressure and demanding tasks that needed to be managed in the municipality. A strong working community was highlighted as essential for managing challenges encountered in daily work.

Berit (FG6): It’s not for anyone to step in and handle all this [light resigned laughter], so there must be competence in place, and a framework (...). We are a team-based service and the expectations regarding how well the team functions together within this framework will be greater, I think.

The somewhat resigned laughter was interpreted as a form of exasperation related to participants’ perceived responsibilities and the unpredictability they encountered. Despite expressing commitment to their work, underlying concern emerged, emphasising the importance of teamwork.

The essential elements of this framework included sufficient competence, adequate staffing, and effective leadership. Leadership was described as critical for addressing changes in the field. Participants noted that shared management strategies were developed within their organisations. Support and understanding from leaders were also emphasised as important for fostering an open workplace culture. This meant that leaders were available for discussion when needed and took participants’ challenges seriously.

Anett (FG2): Being met with understanding for what we are dealing with and having leaders comprehending what we mean when we express frustration or find things difficult, makes one feel more secure in their job as well [mmm ... around the table].

Participants discussed their training in communicating about difficult incidents and experiences. Differences emerged in how this was accomplished, ranging from clear routines and procedures to informal agreements on mutual support. Talking to each other to manage and process feelings related to serious incidents and precarious situations was described as important. This involved collegiality and service users’ involvement in discussions and follow-ups. Participants were committed to addressing difficult incidents and considered the appropriate timing for initiating such conversations with service users. Siv and Lars (FG6) discussed:

Siv: We are quite good at debriefing and going through what we have done and what we have talked about with the service user as well.

Lars: Yes, we involve the service user, then we resolve it, as long as it is possible.

The established systems for education, training, and prevention that contribute to increased confidence in challenging situations were also discussed. The training structure varied: systematic training with structured methods was known but not universally adopted. Specific techniques for managing acute situations were secondary to a preventive perspective, with existing practices described as “nonconfrontational.” De-escalation techniques were highlighted as the most suitable approach for risk reduction and as a core competency for those working in the service. Together with the awareness that violence can occur in municipal mental health services, these factors appeared crucial for collectively creating a sense of security to manage challenges.

## Discussion

In this study, we explored mental health workers’ experiences of workplace violence in municipal mental health services and the strategies they employed in challenging situations. The discussion is structured around two related aspects derived from the four themes. The first section draws primarily on themes 1 and 2, unpredictability and distress, and uncertain collaboration and distribution of responsibility, and considers the consequences of workplace violence. The second section draws primarily on themes 3 and 4, relational and knowledge-based risk reduction, and confidence developed within the workplace, and discusses risk management strategies.

### Consequences of workplace violence

Working conditions within municipal mental health services need to be prioritised. Workplace violence can have significant consequences, including illness and sick leave (Fujimoto et al., 2017; Mento et al., 2020; Tonso et al., 2016). Mental health workers perceived precarious situations as distressing, even in the absence of direct exposure to violent acts. Addressing this is important for mitigating the potential adverse effects on their mental health and well-being (Morse et al., 2012). In addition to defined forms of workplace violence (Konttila et al., 2020; Mento et al., 2020; Tonso et al., 2016; Yang et al., 2018; Zhong & Shorey, 2022), we found that unpredictability and feelings of limited control affect the severity of consequences for mental health workers.

In Norway, safeguarding mental health workers from violent incidents is regulated by the Working Environment Act. Act No. 62 (2005); however, our results suggest that less explicit risk situations are not always addressed systematically. Mental health workers may also lack a vocabulary for articulating the emotional burden associated with the risk of workplace violence. This can contribute to a ‘culture of silence’ (Fowler et al., 2024) and to the normalisation of risk as ‘part of the job’ (Fowler et al., 2024; Parr & Cetrano, 2024). Our results similarly indicate that repeated exposure to unpredictability may become normalised as an unavoidable occupational condition. Violence perpetrated by service users in mental healthcare is frequently excused, potentially promoting tolerance of this phenomenon (Zhong & Shorey, 2022). This tolerance can interact with professional and political ambitions to enhance service users’ rights and autonomy, contributing to the tacit acceptance of workplace violence illustrated in our results.

In recent decades, municipal mental health services have been assigned greater responsibilities and face increasingly demanding tasks. Our results reveal the multifaceted challenges and complex dynamics arising from collaborations among various healthcare levels and agencies. Participants often felt alone in fulfilling their responsibilities, particularly during interactions with service users where there was a risk of violence. This highlights the importance of gaining insight and knowledge about workplace violence from the perspective of municipal mental health services, which differ from frameworks applicable to hospitals (Campbell, 2017; Konttila et al., 2020; Parr & Cetrano, 2024).

We found that municipal mental health workers may work alone, have inadequate access to personnel and alarm systems, and face various challenges related to municipality organisational structure and demographics. Furthermore, the gaps between healthcare levels and agencies can foster unrealistic expectations, which must be addressed. Additionally, we determined that precarious situations may arise from insufficient collaboration. Improving collaboration and facilitating coherent service provision can be achieved through knowledge exchange and improved understanding of each agency’s mandates and services (Elstad et al., 2013; Vik, 2018). Hospital capacity reduction may result in hospitalisations being deprioritized, despite users’ needs. Transforming context-specific challenges into collective concerns and avoiding “blame game situations” in which actors focus on their situations are important (Senneseth & Berg, 2025). Overall, our results indicate a lack of common understanding and suggest that improved collaboration may contribute to ensuring comprehensive services and enhancing mental health workers’ safety.

### Risk management strategies

A common goal in mental health practice is to provide recovery-oriented and person-centred approaches that prioritise service users’ unique processes of living meaningful lives (Jørgensen et al., 2025; Maagerø-Bangstad, 2024). We found that understanding service users and establishing lasting relationships contributed to a dynamic approach to meeting their needs, even in situations involving a risk of workplace violence. Gamme and Eriksson (2018) found that mental health workers’ sensitivity in interactions with service users can help mitigate the risk of violence. Our results suggest that ‘taking chances’ in carefully assessed situations can support service users in managing tense situations. Enduring relationships provided a platform for a dynamic and person-centred approach. Participants were committed to facilitating safety and enabling service users to cope with tense situations. This supports the need to understand a dynamic, person-centred approach in contextual and relational terms rather than relying on manualized and generalised procedures (Hummelvoll et al., 2015; Maagerø-Bangstad, 2024; McCormack & McCance, 2017).

We determined that the confidence necessary to manage precarious situations is cultivated in workplace environments. Confidence serves as a protective factor against negative stressors associated with workplace violence. Internationally, access to mental health professionals presents a substantial challenge, particularly owing to the advanced age of current professionals (Cowman et al., 2017). A substantial proportion of the study participants had extensive experience in mental health services, and five of the six focus groups included participants with over 20 years of experience.

Our results underscore the importance of internal support in navigating emotional and practical challenges. Solidarity and peer support are important for new employees’ learning and their sense of security (Fowler et al., 2024). Additionally, research highlights the necessity of systematic training in preventing and coping with workplace violence (Choi et al., 2023). Addressing challenges associated with workplace violence necessitates ongoing competence development in prevention and management strategies (Cowman et al., 2017; Hagen & Svalund, 2019; Maagerø-Bangstad et al., 2019). We observed diverse strategies that focus on prevention, emphasising the important role of effective leadership as a key premise for fostering competence-building communities. This contrasts with previous results (Parr & Cetrano, 2024) that report negative experiences related to insufficient follow-up and management.

Internationally, interventions to address violence often involve physical coercion, seclusion, or medication (Cowman et al., 2017). However, given the legislative framework governing municipal mental health services, such interventions are neither relevant nor desirable, as they conflict with a dynamic and person-centred approach. To enable mental health workers to provide safe mental healthcare, sufficient staffing and team-based follow-up are preferable. Two-person home visits can enhance mental health worker safety (Choi et al., 2023; Zhong & Shorey, 2022). We found that strong team cohesion and working in pairs were important strategies for managing the risk of violence in municipal mental health services.

### Implications

Our results highlight the importance of reducing the burden associated with the *risk of* workplace violence faced by municipal mental health workers. This consideration is particularly pertinent given the anticipated future shortage of mental health professionals. Our results raise concerns that the strained financial situation within the Norwegian health service sector may affect the provision of mental health services and efforts related to competence development, including training and prevention measures. Research has indicated that workplace violence may affect the quality of support provided to service users (Galinsky et al., 2010; Zhong & Shorey, 2022). Thus, strategies that protect mental health workers and service users must be established, emphasising safety and violence prevention (Cowman et al., 2017).

Workplace violence in municipal mental health services should be examined from various perspectives, particularly focusing on the experiences of service users, managers of municipal mental health services, and additional agencies and healthcare levels. To gain a deeper understanding of the implications for mental health practices, the significance of values concerning workplace violence should also be investigated.

### Methodological considerations

Focus group interviews were used to obtain a comprehensive understanding of participants’ lived experiences of workplace violence in municipal mental health services. We chose focus groups because interaction allowed participants to elaborate on, support, and challenge one another’s accounts, thereby providing insight into shared and divergent understandings (Malterud, 2025). The first author’s extensive practical experience in municipal mental health work was a strength, but it may also have reduced her analytical distance. Collaboration among four authors with different professional backgrounds enabled reflexive dialogue that challenged assumptions while maintaining the study’s focus. Conversations between the moderator and co-moderator after the interviews supported ongoing reflection, while discussions with the advisory board introduced additional perspectives into the research process.

The sample of 38 participants, including seven men, from rural and urban municipalities and two nonprofit organisations represented varied forms of municipal mental health service provision in Norway. All individuals willing to participate were included except at the first author’s workplace. To prevent the first author from influencing the selection, the manager randomly selected participants from the 21 individuals who met the inclusion criteria and volunteered. The results may therefore be transferable to comparable municipal mental health service contexts.

A potential limitation is the absence of more critical perspectives, possibly because mental health workers with strained relationships to the research topic did not participate. The group context may also have created an implicit expectation of consensus. Because workplace violence may evoke shame about one’s own emotional or behavioural reactions, some participants may have moderated or withheld accounts they experienced as professionally uncomfortable. The relative homogeneity of the groups may have facilitated recognition and open discussion among colleagues with shared experience, but it may also have reinforced group norms and constrained dissent (Malterud, 2025). Individual interviews might have provided greater privacy and elicited different nuances. We therefore cannot exclude the possibility that some perspectives were not articulated in the focus group discussions. Conversely, participants elaborated on and sometimes challenged one another’s accounts, and the moderator and co-moderators experienced the group interactions as constructive. Even so, the data reflect what participants were willing and able to share in a group setting.

## Conclusion

Our study found that municipal mental health workers encounter multifaceted challenges, often striving to balance their safety with their responsibility to provide mental healthcare. The potential consequences of workplace violence do not depend exclusively on physical violence; unpredictability and perceived risk also cause distress. This study highlights the need for improved collaboration and shared responsibilities among different healthcare levels and agencies. Familiarity with service users and enduring relationships are essential to a dynamic and person-centred approach that can help prevent and reduce the risk of violence. Together with safety-enhancing measures, such as training and a supportive work environment, these approaches may reduce the extent and consequences of workplace violence. This responsibility must be established in the workplace and anchored within broader institutional structures (Choi et al., 2023). Although only a few municipal mental health service users present a risk of violence, the challenge must be acknowledged because of its potential consequences. Addressing these issues may also enhance the quality of services provided to service users.

## Data Availability

The participants of this study did not give written consent for their data to be openly available.
